# Controlled Release of Biological Control Agents for Preventing Aflatoxin Contamination from Starch–Alginate Beads

**DOI:** 10.3390/molecules24101858

**Published:** 2019-05-14

**Authors:** Jiachang Feng, Jianpeng Dou, Zidan Wu, Dongxue Yin, Wenfu Wu

**Affiliations:** Department of Biological and Agricultural Engineering, Jilin University, Changchun 130000, China

**Keywords:** controlled release, starch, alginate, spore, metalaxyl, kaolin, rice husk powder

## Abstract

For the wise use of fungal biocontrol and metalaxyl fungicide, starch–alginate-based formulations have been developed by encapsulating metalaxyl and non-toxigenic *Aspergillus flavus* spores simultaneously in the form of microspheres using calcium chloride as a cross-linking agent. The formulations were characterized by Fourier transform infrared spectroscopy (FTIR), a scanning electron micrograph (SEM), and thermogravimetry (TGA). Formulation characteristics, including the bead size, entrapment efficiency, swelling ratio of the beads, and rheological properties, were analyzed. The release behavior of beads with different formulations was evaluated. The addition of kaolin and rice husk powder in starch–alginate beads retarded the release profile of spores and metalaxyl. The release of the active ingredient from starch–alginate–kaolin beads and starch–alginate–rice husk powder beads occurred in both a controlled and sustained manner. Additionally, the release rate decreased with the increase of kaolin or rice husk powder content. The beads added with kaolin were slower than the release of rice husk powder. In comparison, spores released slower and lasted longer than metalaxyl. The starch–alginate–kaolin formulations could be used as controlled release material in the field of biocontrol and reduce the harm of fungicides to the environment.

## 1. Introduction

Aflatoxin is a kind of mycotoxin mainly produced by *Aspergillus flavus* or *Aspergillus parasiticus*. Additionally, aflatoxin contamination is a serious problem in many crops, such as corn, peanuts, and other agricultural products, in many countries. Biological control methods have proved to be effective in preventing aflatoxin contamination in several crops, including peanuts, cotton, and corn [1]. Biocontrol strategies were achieved by applying a non-aflatoxigenic strain of *A. flavus* or *A. parasiticus* in soil at a particular time of developing crops. The amount of aflatoxin can be reduced due to the competitive inhibition of a non-aflatoxigenic strain. Abbas et al. proved that the aflatoxin in corn displayed a significant decline after applying the non-aflatoxin producing *A. flavus* strain NRRL30797 [2].

Sterilized barley, paddy, and rice usually act as the carrier of the strain, and the spores are attached to the surface of the grain. After application to the soil surface, these starch-rich grains can absorb moisture and provide an environment for fungal growth and sporulation. Other carriers for fungal biocontrol, including alginate pellets and pre-gelatinized starch–flour granules, have been applied to soil [3]. Recently, a kind of bioplastic containing modified starch was investigated to demonstrate the possibility of replacing cereal grains [4]. The biocontrol strain spores were entrapped in the matrix of the bioplastic. The starch could act as a carbon source during the initial growth of the fungus.

Polysaccharides in nature have attracted considerable attention as controlled release materials [5]. Their strengths are their easy availability, cost effectiveness, and biodegradability. Starch is a renewable and inexpensive raw material for encapsulating, it is completely degradable in nature, and has been utilized to improve the dispersity and stability of microcapsules [6,7,8,9]. Another common polysaccharide in nature, alginate, is obtained from brown macroalgae, and can be developed into a controlled-release formulation after crosslinking with metal ions. In previous studies, a starch–alginate formulation has been investigated for the controlled release of pesticides and the release rate was proportional to both the starch concentration and the sodium alginate concentration [10]. Formulations based on alginate for the slow release of agrochemicals have been developed by adding other materials, including sorbents (bentonite, anthracite, activated carbon) [11], cyclodextrin [12], carboxymethylcellulose [13], pectin [14], chitosan [15], etc.

Agrochemicals including pesticides, herbicides, fungicides, etc., have contributed greatly to crop protection in modern agriculture [10]. In previous research, chemicals were used in various forms in the environment, such as baits, emulsifiable concentrates, sprays, soluble powders, dusts, wettable powders, and so on [16]. Nonetheless, only approximately 10% of the pesticides could achieve their real purpose, and the rest vanished in the environment for various reasons, like evaporation, drifting, leaching, degradation by photolysis, hydrolysis, and microbial activity [17]. An optimal method would be to restrain the amount of agrochemicals released from the formulation in the short term, and minimize residues in the soil and environment. This can be accomplished by encapsulating the chemicals in a controlled-release matrix. The objective of the controlled-release system is to maintain the concentration of the active ingredient so that it ranges between the minimum effective concentration and maximum safe concentration [18]. Recently, there have been a lot of studies on developing materials for delivery systems. These materials contained polymers [19,20], silica [21,22], polyphosphates [23], clay [24], and so on. The polymers, especially in the form of hydrogels and beads, can be critical to controlled-release systems, because they not only provide a sustained release action, but also enhance the soil water holding capacity [25]. Furthermore, they can act as fertilizer in the field after degradation.

Metalaxyl is a widely used acylanilide fungicide with residual and systemic activity against plant diseases caused by the Oomycetes or water-mold fungi [26,27,28]. Usually, its application has included soil incorporation, surface spraying, drenching, soil mix, and seed treatment [29]. Using metalaxyl to prevent and control crop diseases requires it to migrate slowly and release slowly in the soil. In a previous study, Harrison Wanyika experimentally verified the feasibility of embedding metalaxyl with mesoporous silica nanospheres. The results showed that in 30 days, there could be 76% free metalaxyl released in soil, while merely 11.5% and 47% of the metalaxyl entrapped in the nanospheres carrier were released in soil and water, respectively, in the same time. However, the amount of metalaxyl loaded in the nanoparticles was only 14%, and the cost of this material was relatively high for agriculture.

In terms of the existing research, most have focused on the separate encapsulation of the pesticide, bacteria, or fungus. Additionally, literature on the simultaneous and sustained release of fungi and agrochemicals is scanty. Since metalaxyl has often been used on crops such as peanuts and corn that were easily contaminated with aflatoxins [30], it would be more convenient to apply metalaxyl and fungal biocontrol together. Moreover, our preliminary work proved that metalaxyl had no effect on the growth and sporulation of *Aspergillus flavus*. In the present work, we intend to develop starch–alginate-based formulations for the biological control and prevention of aflatoxin by encapsulating metalaxyl and non-toxigenic *Aspergillus flavus* spores simultaneously. Additionally, the feasibility of the formulation as a controlled release substance will be verified. The ingredients in the formulation are starch, alginate, kaolin, and rice husk. All of them are biodegradable, low cost, biocompatible, easily available, and environmentally friendly. Kaolin, as a common clay, has been utilized in controlled-release materials due to its larger surface area and noticeable ion-exchange capacity, and these advantages played a role in absorbing/desorbing the agrochemicals [31]. Rice husk is a universal agricultural waste that is obtained from the shelling of rice. There are large amounts of cellulose and lignin in rice husks [32], which are widely used as delivery systems for bioactive compounds [33]. Therefore, an attempt has been made to develop a sustained release formulation for spores of non-toxic *Aspergillus flavus* and metalaxyl based on starch–alginate.

## 2. Experiment

### 2.1. Materials

Soluble starch (maize starch), sodium alginate, Tween 20, and metalaxyl were obtained from Aladin Ltd. (Shanghai, China). Kaolin was purchased from Xincheng Fine Chemical Co., Ltd. (Shanghai, China). CaCl_2_, KBr, and phosphate buffer solution (PBS) were purchased from Sinopharm Chemical Reagent Co., Ltd. (Shanghai, China). DG18 agar was obtained from Beijing Aoboxing bio-tech Co., Ltd. Rice husk powder came from a local market in Changchun China and passed through a 100-mesh sieve. The non-toxic *Aspergillus flavus* strains (H4-5) were screened in our laboratory, which is located in Changchun China. The water used in the experiment was ultrapure water. All chemicals and reagents were used as received.

### 2.2. Preparation of Spore Suspensions

The isolated non-toxic fungus was grown and maintained on dichloran 18% glycerol (DG18) agar. After incubation for two weeks at 30 °C, spores were removed by gently scraping DG18 plates and suspended in aqueous 0.2% Tween 20. The density of the spore suspension was measured using a hemocytometer and adjusted as described [34].

### 2.3. Preparation of Starch–Alginate-Based Beads

The quantitative starch, alginate, kaolin, or rice husk powder were dispersed in 50 mL of water with heating and magnetic stirring in a water bath. After a homogeneous solution was formed, 5 mg of metalaxyl and 5 mL of spore suspensions were then added to the glue solution and stirred for half an hour. The dispersion was added dropwise by a 50 mL syringe, from a 30 cm height, into 100 mL of 0.1 M CaCl_2_ solution under constant stirring. Thus, the beads were formed and cured for 30 min in the solution. After being taken out of the solution, the beads were washed three times with ultrapure water, and were allowed to dry for 12 hours at 40 °C in a circulating air oven [35]. The compositions of different formula beads are shown in Table 1. The characteristics of starch–alginate beads, such as particle size and entrapment efficiency (%), are listed in Table 2.

### 2.4. Beads Size Measurement

Thirty thoroughly dried samples of each formulation were collected, and their outer diameters were measured using a Vernier caliper. The average bead diameter of each formulation is presented in Table 2.

### 2.5. Characterization

The beads’ formulations were characterized by Fourier transform infrared spectroscopy (FTIR), scanning electron micrograph (SEM), thermogravimetry, and swelling tests. FTIR spectra of the beads were measured with KBr pellets by a Nexus670 FTIR (Thermo Nicolet Corporation, Madison, WI, USA) spectrometer. SEM was taken by HITACHI S-4800 (Hitachi Limited, Tokyo, Japan). The samples were placed on a copper plate and sputtered with gold. Thermogravimetric analysis was achieved under atmospheric pressure using a SHIMADZU DTG-60H Thermal Analyzer (SHIMADZU Corporation, Kyoto, Japan), and the thermal properties of the samples were investigated. Quantitative samples were deposited in a Pt crucible. All experiments employed a linear heating rate of 10 °C/min under N_2_ atmosphere and a flow rate of 50 mL/min, and the temperature ranged from 30 to 700 °C. Swelling properties of the beads were investigated in a buffer solution at 30 °C by monitoring the weight changes during incubation. A certain amount (Wd) of the completely dried beads was immersed in PBS (pH 6.0) and kept at 30 °C. The bloated beads were taken out and instantly weighed (Ws) after the excess of fluid lying on the surfaces was removed by filter paper. The swelling ratio (SR) was obtained by the equation: SR = (Ws − Wd)/Wd. Each group of measurements was repeated three times.

### 2.6. Rheological Properties

The rheological properties of the samples were measured with a small amplitude oscillatory test by an AR500 rheometer (TA Instruments, Fort Sam Houston, TX, USA). After the metalaxyl and spore suspensions were mixed with different starch–alginate-based hydrogels, the samples were carefully transferred into the surface of the lower Peltier plate, and the upper sandblasted plate declined until it reached a 1000 μm gap distance. The storage modulus (G′) and loss modulus (G″) that differed with angular frequency were explored with the parallel plate geometry (diameter = 40 mm). The frequency ranged from 0.1 to 100 rad/s, the amplitude (γ) was 2%, and the temperature was 30 °C.

### 2.7. Release Measurements

#### 2.7.1. Preparation of Metalaxyl Calibration Curve

The calibration curve was prepared with the absorbance of a series of standard solutions of metalaxyl spectrophotometrically by using a SHIMADZU UV-2550 UV-visible spectrophotometer (SHIMADZU Corporation, Kyoto, Japan). The experimental method proposed by Chen et al. [36] has been revised. Five milligrams of pure metalaxyl powder was dissolved in 50 mL methanol to form a reserve solution. The mother liquor was then diluted to different concentrations. Aliquots of a standard solution of metalaxyl were poured into the quartz cuvette. The absorbance of the standard solution was measured at 220 nm (λmax of metalaxyl) against a reagent blank. The absorbance as the horizontal ordinate and the concentration of metalaxyl were taken as the ordinate to obtain the calibration curve.

#### 2.7.2. Encapsulation of Spores and Metalaxyl

It was verified by a pre-experiment that metalaxyl had no effect on the sporulation of non-toxic *Aspergillus flavus* within a certain concentration range. Encapsulation of non-toxic *Aspergillus flavus* spores and metalaxyl was implemented by the process of synthesizing bead formulations, which was stated in Section 2.3.

#### 2.7.3. Entrapment Efficiency (%)

The entrapment efficiency of non-toxic *Aspergillus flavus* spores was measured by the method of a colony-forming unit (CFU/g). In order to determine the spore viability, 0.1 g of beads was dissolved in 10 mL of sodium citrate solution 2.0% (*w/v*) and then gradient dilutions of each sample in 0.85% *w/v* sodium chloride solution were prepared. Aliquots of 100 μL were inoculated on a DG18 medium and cultured for three to five days at 28 ± 2 °C. The results were obtained in terms of colony-forming units per gram of beads. The metalaxyl left in the CaCl_2_ solution from the beads was determined to get the entrapment efficiency of the fungicide. The method is depicted in Section 2.7.1. The entrapment efficiency of spores and metalaxyl is shown in Table 2.

#### 2.7.4. Release of Spores and Metalaxyl

The controlled-release characteristics of starch–alginate-based beads were studied by immersing the dried and loaded samples of each formulation (1 g) in 50 mL of phosphate buffer solution (PBS) at 25 ± 1 °C. The pH of the PBS was adjusted to 6.0 in order to simulate soil acidity. The samples were placed in the dark to avoid the effects of light on matalaxyl.

The concentration of spores in the release medium was measured by the plate count method. After shaking until homogeneous, 200 μL of suspension was taken out and serially diluted in PBS prior to plating. Suspensions were diluted and 100 μL of aliquots were spread on DG18 medium. Plates were incubated at 28 ± 2 °C for three to five days and the results of *Aspergillus flavus* colonies were recorded. After centrifugation at 10,000 rpm, the supernatant was determined by spectrophotometry at 220 nm after every 12 h to get the amount of metalaxyl released. After the sample was removed from the solution, an equivalent amount of fresh medium was poured in to maintain the osmotic pressure stability. All experiments were conducted in triplicate.

#### 2.7.5. Mathematical Modeling of Spores and Metalaxyl Release

To better understand the release process of spores and metalaxyl from the beads, mathematical modeling of the release was applied [37,38,39]. In the existing reports, there was no single model that could perfectly fit all the experimental results of the drug release from polymeric systems [40]. The release kinetics were studied by an empirical formula [38]: $M_{t}/M_{0}=kt^{n}$. Here, $M_{t}/M_{0}$ is the partial release of the active ingredient at time t; ‘k’ is the constant characteristic of the spores, the metalaxyl-polymer system; and ‘n’ is the diffusion exponent of the release mechanism.

### 2.8. Statistical Analysis

Statistical analysis was performed by one way analysis of variance (ANOVA) followed by Duncan’s multiple comparison tests using the software SPSS Statistics 20.0 (IBM Corporation, NY, USA). A value of *p* < 0.05 was taken as the level of significance.

## 3. Results and Discussion

### 3.1. Effect of Formulation Parameters on Characteristics of Starch–Alginate Beads

The components of starch–alginate beads are shown in Table 1. The dry weight of beads increased with the increase of added contents (kaolin or rice husk powder). This might be attributed to the decrease in leaching of the reaction mixture in the beads’ synthesis process. The diameters of the beads varied from 1.95 ± 0.10 mm to 2.37 ± 0.09 mm. It was shown that the size of the beads increased when increasing the added content, and the rice husk powder had a greater effect on the size of spheres than kaolin (Table 2). Spores and metalaxyl were embedded in starch–alginate beads, and the entrapment efficiency ranged from 44.31 ± 3.03% to 84.55 ± 1.56% and 64.00 ± 2.59% to 86.63 ± 1.78% for spores and metalaxyl, respectively (Table 2). It was observed that the formulation parameters had no effect on the entrapment efficiency (*p* > 0.05). This may be due to the constant feed of conidial suspension and metalaxyl when synthesizing different formulations [10]

### 3.2. Characterization

#### 3.2.1. FTIR Spectroscopy Analysis

**Figure 1:**
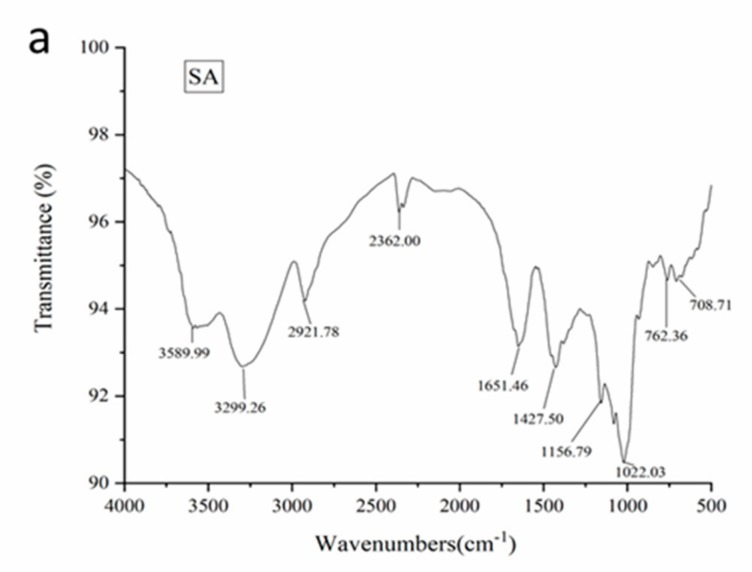

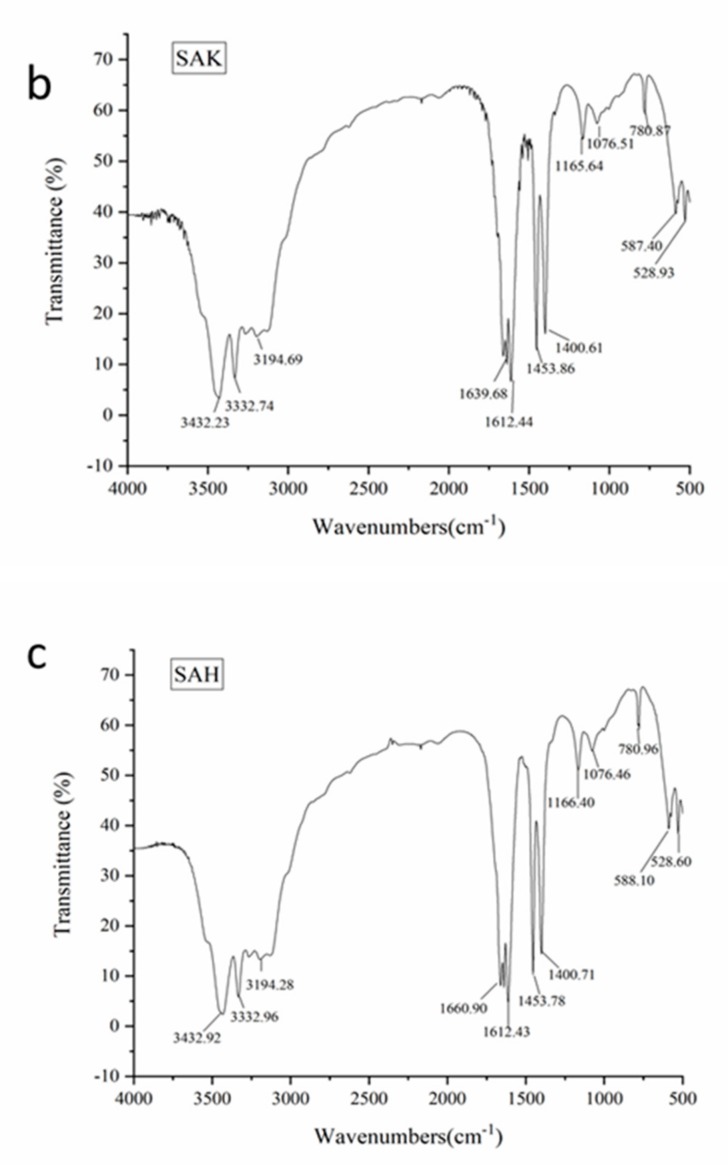
FTIR spectra of starch–alginate based beads: (**a**) starch-alginate beads (**b**) starch-alginate-kaolin beads (**c**) starch-alginate-rice husk powder beads.

FTIR spectra of the samples were measured with KBr pellets and are presented in Figure 1a–c. In all three cases, the FTIR peak appeared at wave numbers between 3200 and 3600 cm^−1^. This is related to the –OH group together with some complex bands in the region 1200–1030 cm^−1^ due to C-O, C-H, and C-O-C stretching vibrations, which are the characteristic of natural polysaccharides. Furthermore, the absorption bands near 780 cm^−1^ are due to vibrational modes of pyranose rings of polysaccharides that have been espied [10,41]. The peak at 1453 cm^−1^ was due to the stretching in aromatic rings [41]. The stretching absorption band around 1651 cm^−1^ was observed in all three figures due to the existence of carboxylate anions of calcium alginate [42]. The absorption peak at 1400 cm^−1^ in three cases was due to the presence of the C-N bond, which belonged to metalaxyl. This proved that metalaxyl was entrapped in the beads.

#### 3.2.2. SEM Analysis

**Figure 2:**
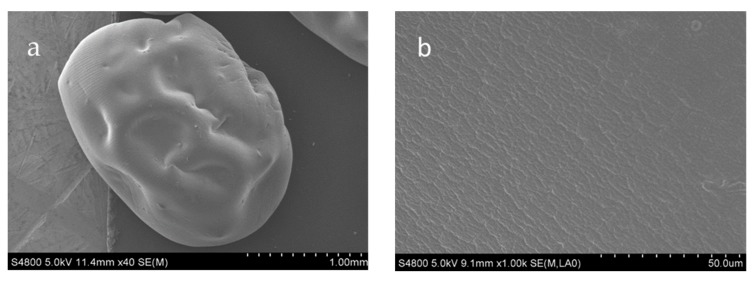

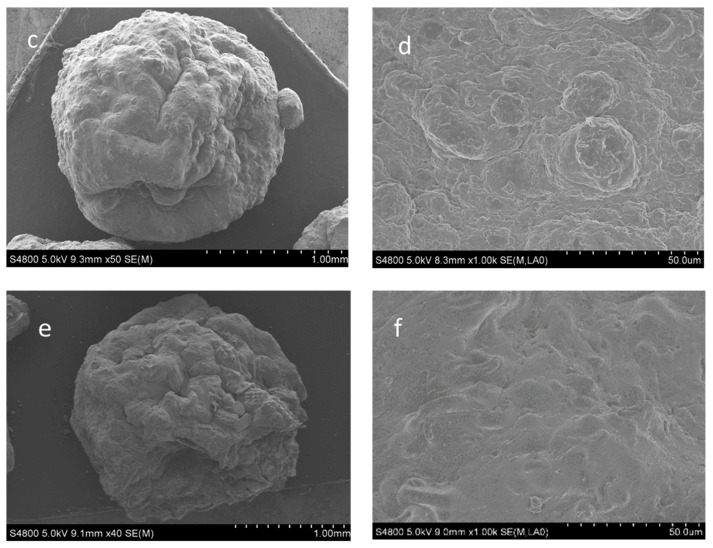
Scanning electron micrographs of starch-alginate based beads: (**a**,**b**) Starch–alginate beads at different magnifications {a × 40; b × 1000}. (**c**,**d**) Starch–alginate–kaolin beads at different magnifications {c × 50; d × 1000}. (**e**,**f**) Starch–alginate–rice husk powder beads at different magnifications {e × 40; f × 1000}.

It was observed that all beads were ellipsoidal shapes by SEM (Figure 2a–f). Observation at low magnification (×40, ×50) showed that the surfaces of SA (starch-alginate) beads were smooth, whereas SAK (starch-alginate-kaolin) beads and SAH (starch-alginate-rice husk powder) beads’ surfaces were rough and had a high porosity. This might be attributed to the addition of kaolin or rice husk powder, where the particles presented a great deal of steps, kinks, and broken edges. Furthermore, this may be influenced by the drying process, as the hydrogel part contracted substantially by losing water, whereas the hydrophobic part did not change dimensions during drying [43].

The shape of SA was more oval-shaped, while SAK and SAH were closer to a sphere. This was different from Baljit Singh’s results, which stated that the SA bead had a spherical shape and became elliptical with the incorporation of clay [44]. This may be related to the decrease of viscosity of starch–alginate hydrogel with the addition of kaolin or rice husk powder, which was verified by the rheological properties. A higher viscosity solution will lead to elongated droplets during extrusion, and the droplets could not recover a spherical shape before contacting the curing solution [43]. At a higher magnification (×1000), all beads had glossy and flat surfaces.

#### 3.2.3. Thermogravimetric Analysis

**Figure 3:**
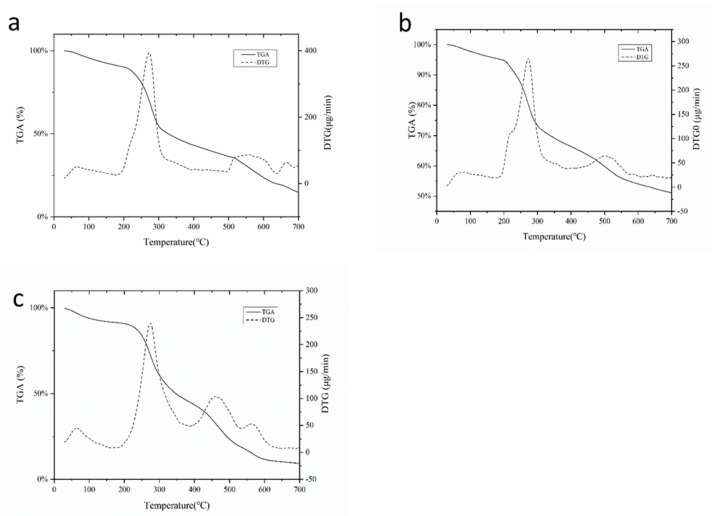
Thermogram of starch–alginate based beads: (**a**) starch-alginate beads (**b**) starch-alginate-kaolin beads (**c**) starch-alginate-rice husk powder beads.

The thermal stability of beads was investigated by thermogravimetry analysis (TGA) and derivative thermogravimetry (DTG), which is shown in Figure 3. Generally speaking, thermal decomposition has several processes, including the desorption of physically absorbed water and gases, loss of structural water (dehydration), and depolymerization along with the rupture of C-C and C-O bonds in the ring units leading to the evolution of CO, CO_2_, and H_2_O, which eventually formed polynuclear aromatic and graphitic carbon structures [45,46]. In the present study, all three cases showed similar trends in the first stage of thermal decomposition (Figure 3a–c). In the TGA curves, the loss of different forms of water molecules as the temperature ranged from 30 to 200 °C was due to their interaction with the polysaccharides [47]. The initial decomposition temperatures of SA, SAK, and SAH beads were 239.02 °C, 221.15 °C, and 233.98 °C, respectively. The addition of kaolin and rice husk powder raised the thermal stability of the beads. Further, the incorporation of kaolin was better than the rice husk powder for the thermal stability of the formulation. This could be attributed to the good thermal stability of kaolin and the interaction between kaolin particles and polysaccharide molecules [48,49]. Similar trends have been observed in other studies [50]. In each case, the remaining residue at the end was 14.63%, 51.07%, and 9.25%, for SA, SAK, and SAH, respectively. There was more remaining residue of SAK beads due to the presence of oxides, carbonates, and silicates [44].

#### 3.2.4. Swelling Ratio

**Figure 4:**
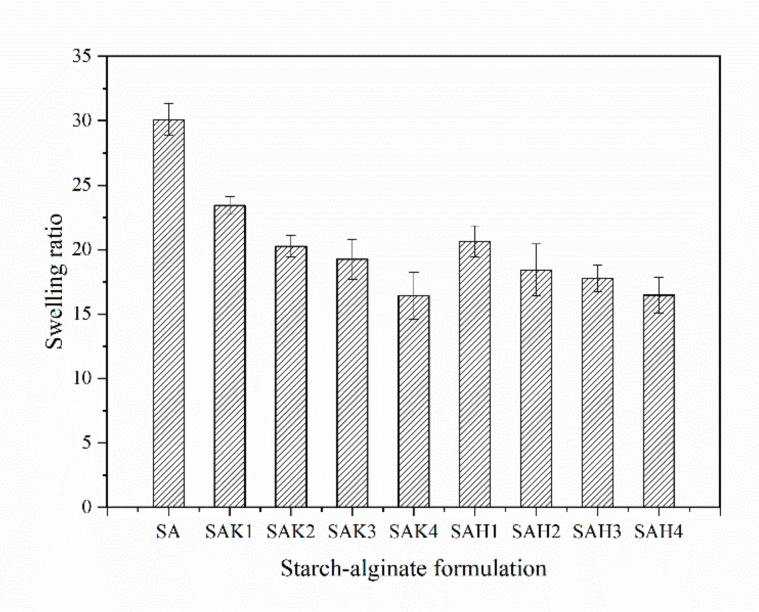
Swelling ratio of beads with different formulations in PBS at 30 °C.

The influence of kaolin and rice husk powder on the swelling ratio of starch–alginate beads after incubation in PBS for 24 h is presented in Figure 4. The swelling process can be explained by filling the void regions of the polymer network and the center of the larger pores and macropores after absorbing free water and binding water [51]. The addition of kaolin or rice husk powder in starch–alginate beads decreased the swelling ratio, and the more kaolin or rice husk powder was added, the smaller the swelling ratio (Figure 4). The starch–alginate beads showed a swelling ratio of 30.1, which decreased with the combination of kaolin 23.43 (SAK1) and rice husk powder 20.65 (SAH1) in the formulation. Additionally, the swelling ratio of SAK4 was 7.01 lower than that of SAK1, and SAH4’s swelling ratio was 4.17 lower than SAH1. The reason for this may be that the kaolin or rice husk powder particle served as a filler and physically filled in the network of beads, and the amount of hydrophobic groups increased with the increase in the amount of kaolin or rice husk powder, which reduced the water absorption capacity [52].

#### 3.2.5. Rheological Properties

**Figure 5:**
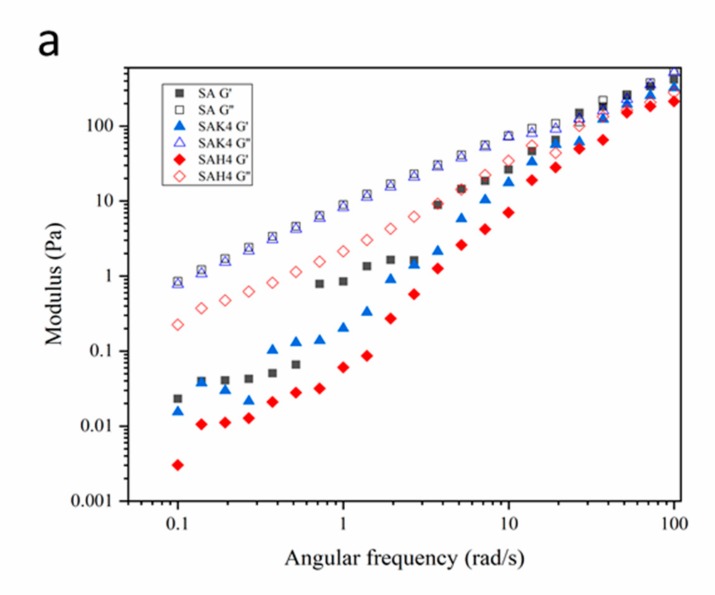

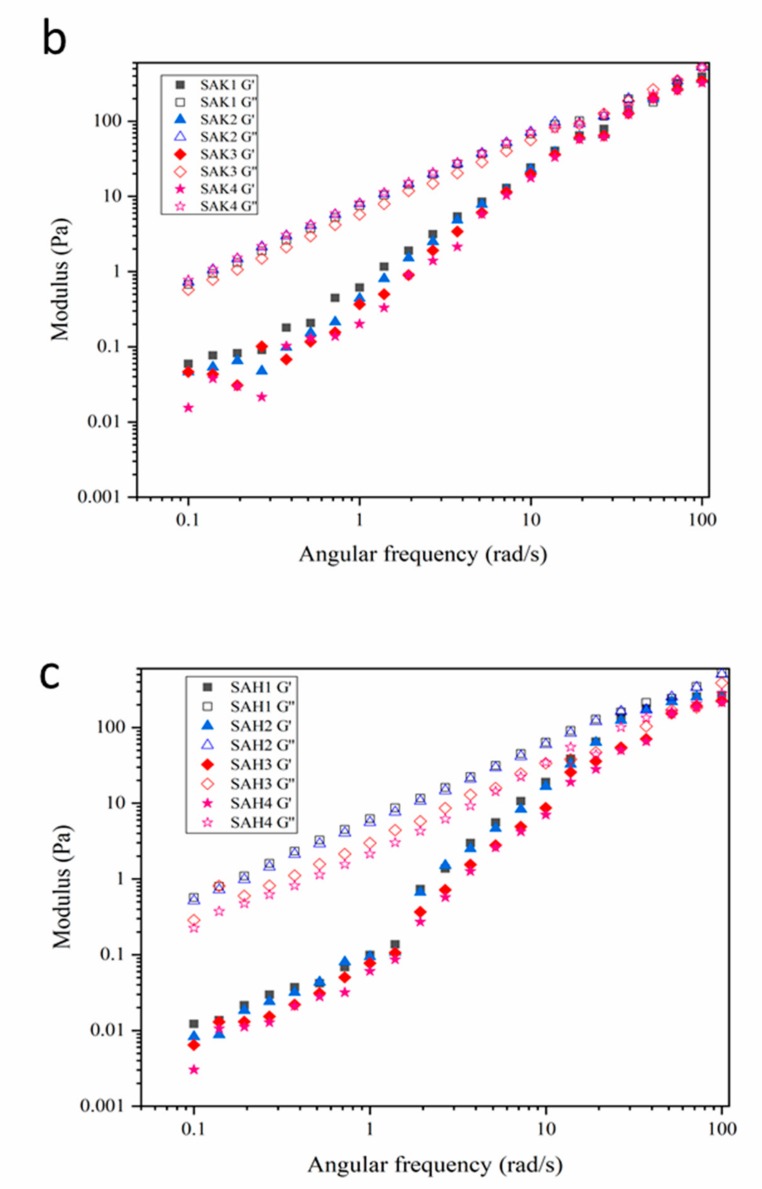
Frequency dependence of storage modulus (G′) and loss modulus (G″) of beads: (**a**) with different kaolin contents (**b**) with different rice husk powder contents (**c**) with different formulations.

The rheological properties of the composite gel were determined by measuring the storage modulus (G′) and loss modulus (G″) as a function of angular frequency at 30 °C (Figure 5). In nearly all cases, the G′ and G″ increased with the increase of angular frequency, and the G″ values were larger than the corresponding G′ values, which means the composite gel has a better viscosity than elasticity [53], verifying the typical liquid viscosity behavior. Both G′ and G″ decreased slightly with the increase in the amount of kaolin or rice husk powder (Figure 5b,c); furthermore, they were both smaller than those of the starch–alginate hydrogel (Figure 5a). This might be due to the amount of gum that decreased with the increase of kaolin or rice husk powder. In addition, the viscosity of the starch–alginate-based solution was related to the shape of the dried beads, compared with the results of SEM. Such rheological behaviors of the complex gel indicated that these materials are suitable as a wall material with a good flexibility and mechanical strength for efficient encapsulation [54].

### 3.3. Release of Spores and Metalaxyl

**Figure 6:**
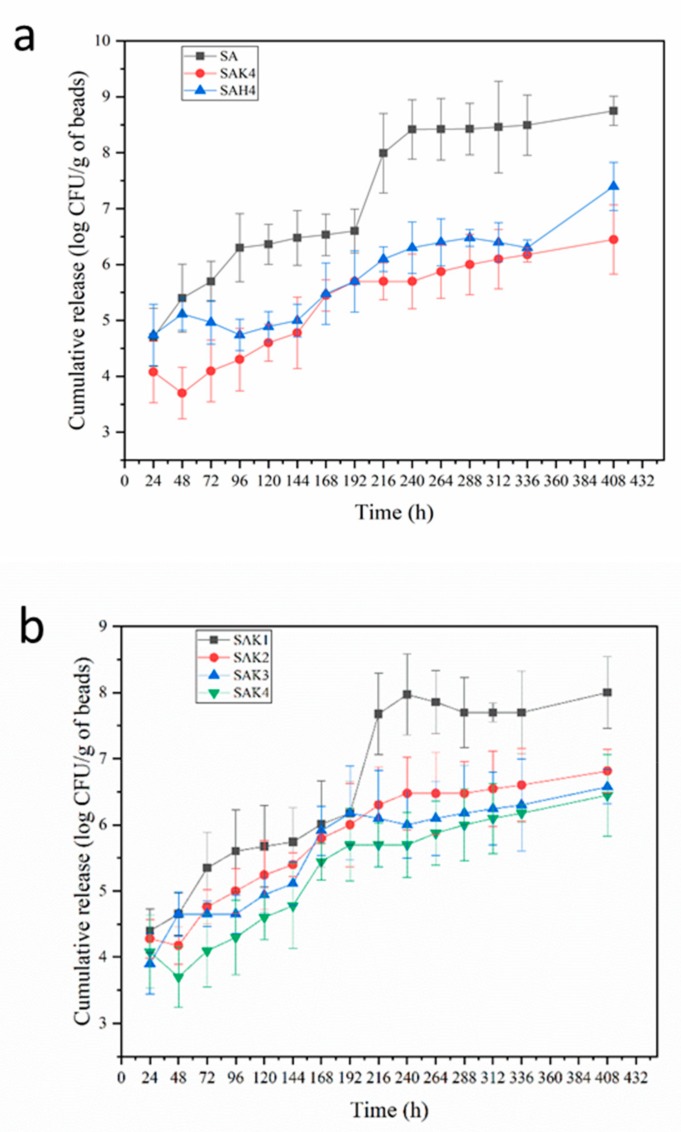

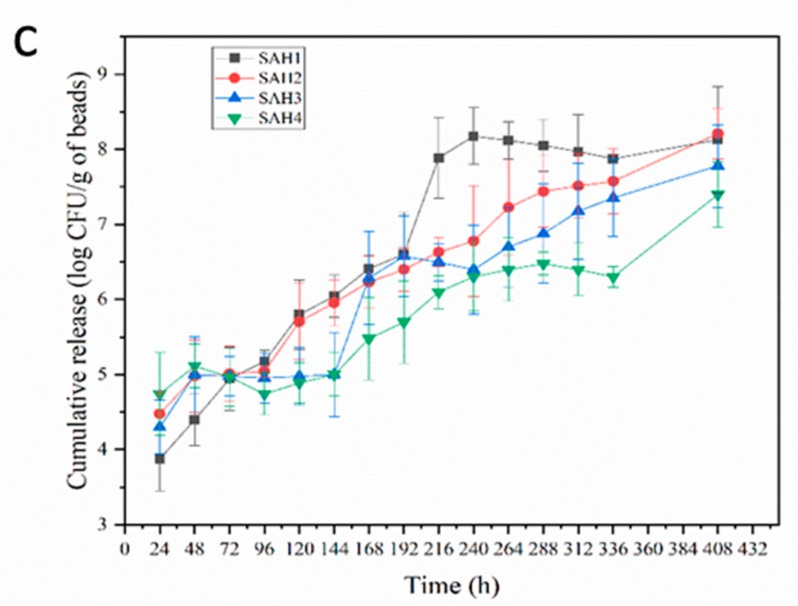
Cumulative release of spores from starch–alginate based beads: (**a**) With different formulations (**b**) With different kaolin contents (**c**) With different rice husk powder contents.

**Figure 7:**
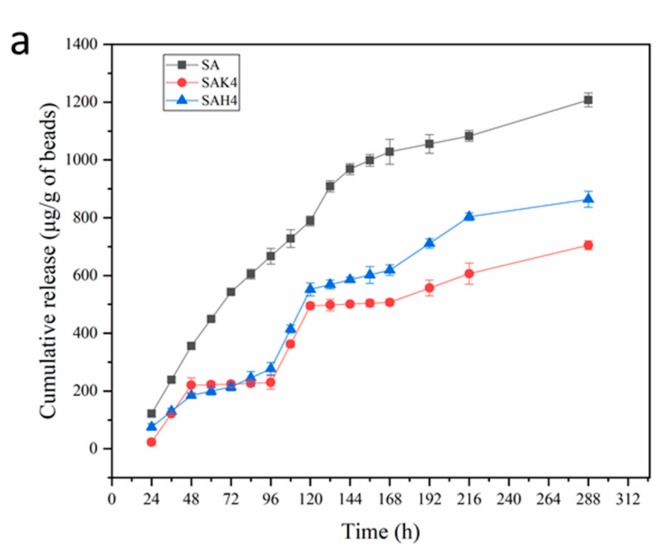

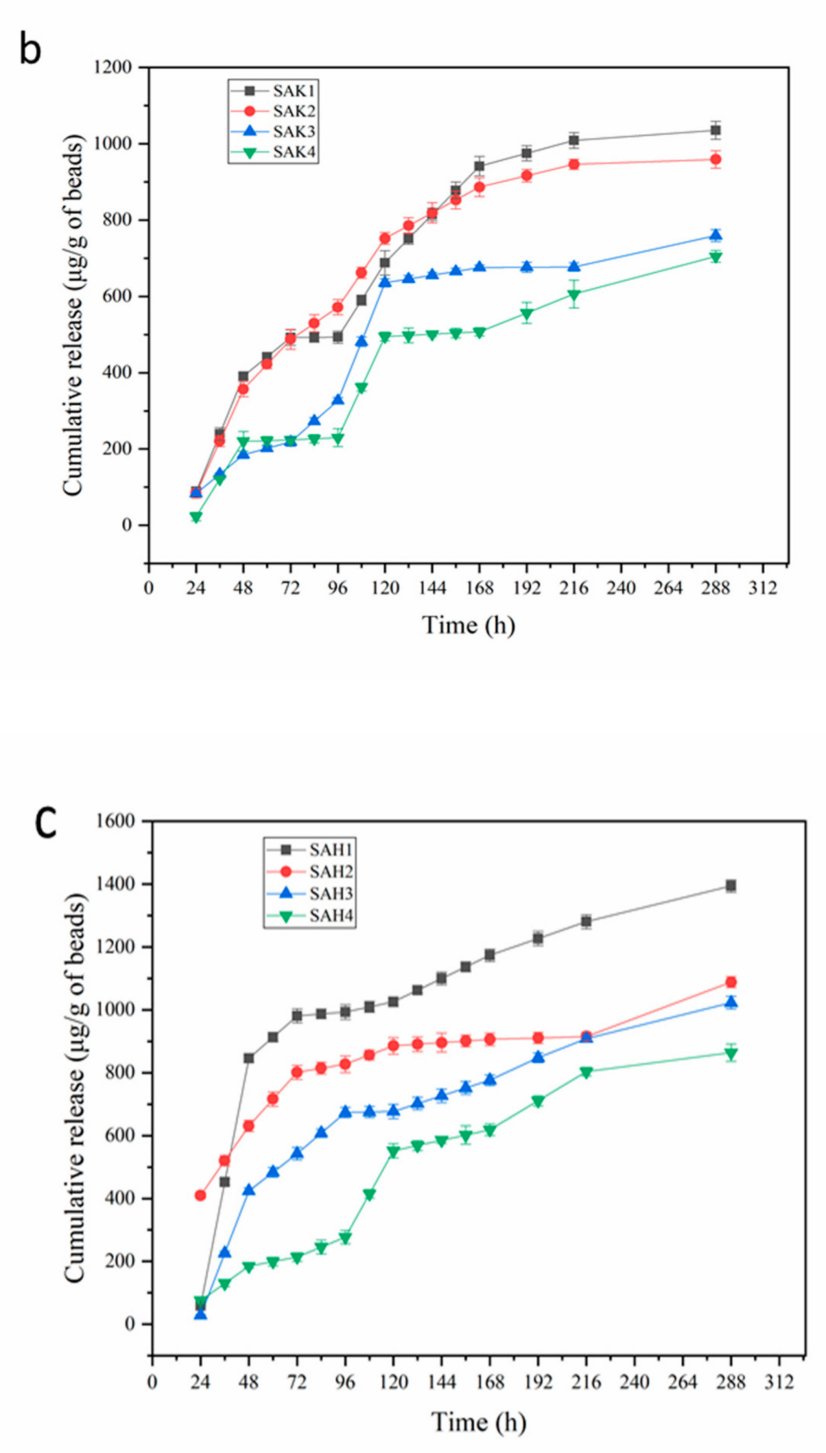
Cumulative release of metalaxyl from starch–alginate based beads: (**a**) With different formulations (**b**) With different kaolin contents (**c**) With different rice husk powder contents.

The release of the polymer-encapsulated substance undergoes two processes; first, the releasing medium penetrates the network of polymers and swells, followed by diffusion along the aqueous pathways to the surface of the granules. In the present study, the dried and loaded beads were immersed in PBS (pH 6.0) at 25 ± 1 °C in the light-proof condition, to simulate the soil environment. The release of spores and metalaxyl from the beads of different formulations has been investigated at the interval of 24 h and 12 h for 400 h and 300 h, respectively. The results are depicted in Figure 6 and Figure 7.

It is obvious from the figures that the release of both spores and metalaxyl from the beads increased with time. The maximum release of spores from SA beads was 8.49 log CFU mL^−1^ after 336 h, whereas, with the addition of rice husk powder and kaolin, the amount of released spores decreased to 6.30 (SAH4) and 6.17 (SAK4) log CFU mL^−1^, respectively (Figure 6a). Further, the release of spores from starch–alginate (SA) beads at 408 h had not increased substantially. However, the release of spores continued at 408 h in the presence of rice husk powder (SAH4) and kaolin (SAK4). The release rate decreased with the increase of rice husk powder or kaolin’s concentration, SAH4 released 97.3% fewer spores than SAH1, and SAK4 released 97.05% less spores than SAK1 after 366 h (Figure 6b). It is observed that the release of spores from the beads was carried out in a controlled and sustained manner, which was exactly what biocontrol requires, as the fungal biocontrol needs to survive longer in the environment.

The cumulative release of metalaxyl from starch–alginate-based beads is shown in Figure 7. As demonstrated for the release of spores, the release rate of metalaxyl from SA was the quickest; nevertheless, the release of metalaxyl from starch–alginate–rice husk powder (SAH4) and starch–alginate–kaolin (SAK4) was much slower. The amount of released metalaxyl in the medium from SA after 216 h was 34.77% and 78.59% higher than that of SAH4 and SAK4, respectively. In three cases, the release of metalaxyl extended after 216 h, according to the data at 288 h. This means that the release of metalaxyl was also sustainable and controllable, which is the primary requirement for the utility of agrochemicals to restrain the environment and health hazard [10,18]. Consistent with the release of spores, the higher the rice husk powder or kaolin concentration, the slower the release rate of the formulation. After 216 h, the cumulative released metalaxyl of SAH1 was 1280.66 μg/g beads, while that of SAH4 was 803.48 μg/g beads; similarly, SAK1 was 1008.76 μg/g beads and SAK4 was 606.36 μg/g beads (Figure 7b,c).

The sustained release when increasing the content of rice husk powder or kaolin might be attached to the better adsorption of spores and metalaxyl on the additions’ particles. Jianfa Li stated that a higher clay percentage induces a dense and viscous suspension, which would give tailed hydrogel beads in gelation [55]. According to Gerstl. Z’s observation, the bead radius has a great effect on the release rates; the smaller the radius, the faster the release [56]. This is confirmed by the release behavior and the particle size of different formulations of beads (Table 2). In addition, it is related to the amount of spores and metalaxyl loaded in the beads of different formulations. These results were in accordance with the swelling experiments. Similar results have been recorded in other literature [51,55,56,57].

Compared with the release of spores, the release of metalaxyl was faster in the early stage and slower in the later stage. Moreover, spores’ release lasted longer (Figure 6a and Figure 7a). This might be due to the larger size of spores, and the dissolution of metalaxyl in the medium would result in faster release.

To investigate the mechanisms of spores and metalaxyl release from the starch–alginate beads, the diffusion characteristic constant ‘k’ and diffusion exponent ‘n’ were obtained by calculating the intercept and slope of the plot of ln (*M_t_/M_0_*) versus ln t. The values of ‘n’ and ‘k’ and the diffusion mechanism for the release dynamics of spores and metalaxyl were evaluated and the results are presented in Table 3 and Table 4. In terms of spores’ release, all values of the diffusion exponent ‘n’ were <0.5, and the mechanistic aspects of the process were finely described by Normal Fickian’s equation. As for the release of metalaxyl, the diffusion exponent ‘n’ was not regular and the value of ‘n’ for SA, SAK1, SAK2, SAH1, was 0.5–1, while SAH3 and SAH4 > 1 and SAH2 < 0.5. Thus, the mechanism is Non-Fickian for SA, SAK1, SAK2, and SAH1; Case II type for SAH3 and SAH4; and Normal Fickian for SAH2 [37,38,39].

## 4. Conclusions

Starch–alginate-based beads were successfully developed by the ionic gelation method using calcium chloride as a crosslinking agent. Spores of a non-aflatoxigenic strain and metalaxyl were encapsulated in the beads confirmed by the measurement of entrapment efficiency and FTIR. It is concluded that the release of spores and metalaxyl from the beads was controllable and sustainable, which is important in the biocontrol of aflatoxin and management of pesticides’ harm to the environment and ecosystem.

The attendance of kaolin and rice husk powder in starch–alginate-based beads made the release rate slower, which prolonged the release of spores and metalaxyl and enhanced the bioavailability of the formulation. Moreover, the increase in kaolin or rice husk powder contents in these constitutions decreased the release of spores and metalaxyl. Further, the release rate of starch–alginate–kaolin beads was slower than that of rice husk powder-containing formulations. The spores’ release kinetics were consistent with the Normal Fickian diffusion mechanism, while the release of metalaxyl from most of the formulations occurred through the Case II diffusion mechanism. Thus, it can be concluded that the use of kaolin and rice husk powder as an addition to the starch–alginate-based encapsulation systems is very effective for the retarded release of bioactive substances. The starch–alginate–kaolin or starch–alginate–rice husk powder beads could be considered carriers of fungal biocontrol in the field of biological control and prevention, and also have the effect of a slow release of pesticides. In view of the slow release rate, starch–alginate–rice husk powder beads are suitable for use from the beginning of crop growth, while the starch–alginate–kaolin beads are suitable for later use, starting from the key stages of crops affected by *Aspergillus flavus*, such as flowering or podding stages.

## Figures and Tables

**Table 1:** Formulation parameters for the synthesis of starch–alginate beads, for which the amount of metalaxyl and the concentration of CaCl_2_ solution in each formulation was 5 mg and 0.1 M, respectively.

Sample	Starch (% *w/v*)	Alginate (% *w/v*)	Kaolin (% *w/v*)	Rice Husk Powder (% *w/v*)	Beads Weight (g)
SA	10	1.5	-	-	1.76
SAK1	10	1.5	1	-	2.16
SAK2	10	1.5	2	-	2.53
SAK3	10	1.5	3	-	2.73
SAK4	10	1.5	4	-	3.00
SAH1	10	1.5	-	1	2.17
SAH2	10	1.5	-	2	2.60
SAH3	10	1.5	-	3	2.57
SAH4	10	1.5	-	4	2.78

**Table 2:** Characteristics of starch–alginate beads.

Sample	Bead Diameter (mm)	Entrapment Efficiency of Spores (%)	Entrapment Efficiency of Metalaxyl (%)
SA	1.95 ± 0.10	61.75 ± 1.25	79.57 ± 1.66
SAK1	2.02 ± 0.06 ^a,^*	48.56 ± 2.33 ^c^	86.63 ± 1.78 ^a^
SAK2	2.03 ± 0.08 ^a^	63.17 ± 0.84 ^a^	76.39 ± 3.32 ^b^
SAK3	2.10 ± 0.07 ^a^	44.31 ± 3.03 ^c^	74.89 ± 2.85 ^b^
SAK4	2.10 ± 0.07 ^a^	56.34 ± 2.64 ^b^	77.58 ± 1.96 ^b^
SAH1	2.08 ± 0.07 ^c^	76.09 ± 1.88 ^c^	73.34 ± 1.67 ^b^
SAH2	2.19 ± 0.04 ^b,c^	84.55 ± 1.56 ^a^	85.05 ± 3.48 ^a^
SAH3	2.27 ± 0.06 ^a,b^	67.40 ± 2.54 ^b^	64.00 ± 2.59 ^c^
SAH4	2.37 ± 0.09 ^a^	83.42 ± 2.48 ^a^	67.56 ± 2.63 ^c^

* Mean ± standard deviation with different letters indicates significant differences between formulations using Duncan’s multiple range tests. ^a, b, c^ Different letters in the same series indicate significant difference at *p* < 0.05.

**Table 3:** Diffusion constants and mechanism involved in the release process of spores from different formulations of beads.

Formulation	Equation	n	K	Mechanism
SA	Y = 0.2395X − 1.4731	0.24	0.23	Normal Fickian
SAK1	Y = 0.2468X − 1.5782	0.25	0.21	Normal Fickian
SAK2	Y = 0.2000X − 1.4683	0.20	0.23	Normal Fickian
SAK3	Y = 0.1904X − 1.4312	0.19	0.24	Normal Fickian
SAK4	Y = 0.2121X − 1.6105	0.21	0.20	Normal Fickian
SAH1	Y = 0.3075X − 1.9013	0.30	0.15	Normal Fickian
SAH2	Y = 0.2247X − 1.5118	0.22	0.22	Normal Fickian
SAH3	Y = 0.2146X − 1.4968	0.21	0.22	Normal Fickian
SAH4	Y = 0.1512X − 1.2319	0.15	0.29	Normal Fickian

**n**—diffusion exponent, **k**—diffusion characteristic constant.

**Table 4:** Diffusion constants and mechanism involved in the release process of metalaxyl from different formulations of beads.

Formulation	Equation	n	K (×10^−3^)	Mechanism
SA	Y = 0.9011X − 5.4281	0.90	4.39	Non-Fickian
SAK1	Y = 0.8969X − 5.4140	0.89	4.45	Non-Fickian
SAK2	Y = 0.9027X − 5.1622	0.90	5.73	Non-Fickian
SAK3	Y = 1.0009X − 5.9081	1.00	2.72	Case II
SAK4	Y = 1.1495X − 6.7615	1.15	1.16	Case II
SAH1	Y = 0.8603X − 4.6581	0.86	9.48	Non-Fickian
SAH2	Y = 0.3416X − 2.3020	0.34	100.04	Normal Fickian
SAH3	Y = 1.0525X − 5.7209	1.05	3.28	Case II
SAH4	Y = 1.0473X − 6.0509	1.05	2.36	Case II

**n**—diffusion exponent, **k**—diffusion characteristic constant.
